# Size‐based protocol optimization using automatic tube current modulation and automatic kV selection in computed tomography

**DOI:** 10.1120/jacmp.v17i1.5756

**Published:** 2016-01-08

**Authors:** Robert D. MacDougall, Patricia L. Kleinman, Michael J. Callahan

**Affiliations:** ^1^ Department of Radiology Boston Children's Hospital Boston MA USA

**Keywords:** computed tomography, pediatric, protocols, size‐specific dose estimate, diagnostic reference ranges

## Abstract

Size‐based diagnostic reference ranges (DRRs) for contrast‐enhanced pediatric abdominal computed tomography (CT) have been published in order to establish practical upper and lower limits of CTDI, DLP, and SSDE. Based on these DRRs, guidelines for establishing size‐based SSDE target levels from the SSDE of a standard adult by applying a linear correction factor have been published and provide a great reference for dose optimization initiatives. The necessary step of designing manufacturer‐specific CT protocols to achieve established SSDE targets is the responsibility of the Qualified Medical Physicist. The task is straightforward if fixed‐mA protocols are used, however, more difficult when automatic exposure control (AEC) and automatic kV selection are considered. In such cases, the physicist must deduce the operation of AEC algorithms from technical documentation or through testing, using a wide range of phantom sizes. Our study presents the results of such testing using anthropomorphic phantoms ranging in size from the newborn to the obese adult. The effect of each user‐controlled parameter was modeled for a single‐manufacturer AEC algorithm (Siemens CARE Dose4D) and automatic kV selection algorithm (Siemens CARE kV). Based on the results presented in this study, a process for designing mA‐modulated, pediatric abdominal CT protocols that achieve user‐defined SSDE and kV targets is described.

PACS numbers: 87.57.Q‐, 87.57.qp, 87.53.Bn

## I. INTRODUCTION

Recent studies have taken a rigorous approach to define size‐based diagnostic reference ranges (DRRs) for contrast‐enhanced, computed tomography (CT) examinations of the abdomen and pelvis. These DRRs, derived from radiologist scoring of studies across multiple institutions,^(1)^ form the basis of a practical method for determining a target size‐specific dose estimate (SSDE) for all patient sizes by scaling the SSDE of a standard adult using a correction factor (CF). This approach is recommended by the Image Gently® campaign.^(2)^ Such efforts aim to provide practical recommendations for striking a balance between absorbed radiation dose and diagnostic confidence of the interpreting radiologist.

Once size‐based SSDE levels have been established, it is the job of the Qualified Medical Physicist (QMP) to assist in the design and implementation of protocols that achieve the defined SSDE target for each patient size. This task is straightforward if fixed‐mA protocols are used since SSDE scales linearly with mA for a given patient size. Using fixed mA techniques, results are predictable and consistent. However, this approach fails to utilize the dose saving potential of automatic exposure control (AEC) algorithms,^(3)^ such as on‐the‐fly mA‐modulation and adaption to patient geometry and density.^(4)^ AEC is particularly useful when imaging body parts with relatively large differences in attenuation on a single study, such as CT examinations of the chest, abdomen or pelvis.

Physicists wishing to take advantage of AEC algorithms provided by CT scanner manufacturers are forced to deduce the operation of the AEC algorithm from technical documentation, published literature or extensive phantom testing for a wide range of patient sizes. When image quality parameters are defined in terms of kV and effective mAs to a reference patient (e.g., reference kV and quality reference mAs), it can be difficult to predict the effect these algorithms will have on patients that vary significantly in size and shape compared to the reference patient. It is the opinion of the authors that technical documentation should include kV and output data from testing on standard phantoms for all available AEC parameters. This is not currently the standard and as such, without available phantoms and testing time, physicists must design protocols in the absence of empirical data regarding the dose delivered to pediatric patients of various sizes in a clinical setting.

The protocol optimization process is also complicated by a trend toward increased automation in technique selection (both mA and kV) in an effort to ostensibly minimize user error and improve image quality and consistency. The result, however, is that minor changes to a protocol defined for a reference patient can have unintended, adverse effects on dose and image quality for small and large patients. The optimization process can become a repetitive, time‐consuming, and often frustrating task for the physicist and clinical staff. Safety concerns may also arise when techniques have not been carefully designed prior to actual patient scanning, raising issues of suboptimal image quality and/or exposure to unnecessary radiation and violating the ALARA principle.

In this study, we aim to characterize the user‐controlled parameters within a single‐manufacturer AEC algorithm in terms of the effect on SSDE and automatic kV selection across a wide range of phantom sizes. These data are an example of what could be provided by the manufacturer in technical documentation to assist the physicist in designing protocols that achieve size‐based dose targets. Based on the results, we describe a process for building size‐specific pediatric protocols, utilizing measured patient thickness, to achieve size‐specific SSDE and kV targets.

## II. MATERIALS AND METHODS

This study used anthropomorphic phantoms to model a single‐manufacturer AEC algorithm. The research plan was to model the effect of user‐controlled parameters within Siemens CARE Dose4D and CARE kV in terms of SSDE and automatic kV selection. Using the empirical model developed from phantom testing, we describe a process for building a set of size‐specific CT protocols that consistently achieve SSDE and kV targets, using published guidelines^(1)^ as an illustrative example.

### A. Anthropomorphic phantoms

**Figure 1:**
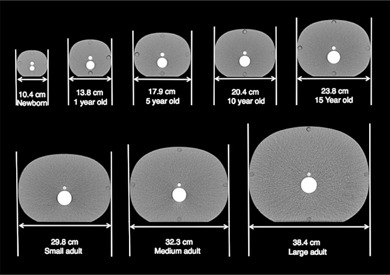
Axial CT images of anthropomorphic phantoms ranging in size from newborn to large adult. All phantoms contained a spinal insert (large insert) and a simulated vessel (small insert) filled with 6 mg/cc iodine contrast (170 HU).

Eight anthropomorphic phantoms (Model 007TE, Computerized Imaging Reference Systems, Inc., Norfolk VA), ranging in size from newborn to large adult, were used to simulate a wide range of abdomen sizes. All phantoms were constructed with soft tissue‐equivalent epoxy and a bone insert to simulate the spine. Additionally, center and peripheral holes (1 cm diameter) were available for insertion of a contrast rod. Water‐equivalent rods were placed into all peripheral holes and a contrast rod (6 mg/cc iodine contrast [170 HU at 120 kV]) was placed into the center hole to simulate contrast injection. Reconstructed axial CT images of the phantom set are shown in Fig. 1. The antero–posterior (AP) and lateral dimensions along with the effective diameter of each phantom size can be found in Table 1. Effective diameter was calculated from Table 1A of the American Association of Physicists in Medicine (AAPM) Task Group (TG)

**Figure 2:**
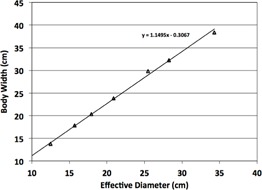
Fit of body width (BW) vs. effective diameter of anthropomorphic phantoms.

Report 204.^(5)^ In addition, body width (BW), taken as the lateral dimension of each phantom, was plotted against effective diameter for each phantom size in Fig. 2. The trend line equation was used to convert effective diameter to BW for comparison with published DRRs,^(1)^ which used BW as the size metric.

**Table 1:** Anthropomorphic abdomen phantom sizes by nominal age.

*Age*	*AP (cm)*	*LAT (cm)*	$D_{\text{Eff}}\;(\text{cm})$
Newborn	8.89	10.42	9.62
1 year	11.28	13.78	12.47
5 years	13.80	17.85	15.70
10 years	15.78	20.36	17.92
15 years	18.26	23.85	20.86
Small adult	21.75	29.85	25.48
Medium adult	24.72	32.26	28.24
Large adult	30.66	38.41	34.32

AP = anteroposterior dimension; LAT = lateral dimension; $D_{\text{EFF}}$ = effective diameter.

### B. CT scanner hardware and software

The CT component of a Siemens Biograph mCT•S (32 detector row, 40 detector electronic channels) PET‐CT scanner (Siemens Medical Solutions USA, Inc. Knoxville, TN; Manufactured, 2012, Software version VG40C) was used to scan all phantoms with different configurations of user‐adjustable parameters in order to fully characterize the AEC software. However, we did find limitations in this software version with respect to kV selection which are mentioned in the limitations section in Appendix A.

There are two components to the AEC software: Siemens CARE Dose4D and CARE kV. CARE Dose4D is the mA‐modulation algorithm and CARE kV is the automatic kV selection algorithm. Though both components are closely related, we will separate them in this discussion for the purpose of describing the function of individual parameters.

### C. CARE Dose4D overview

There are three components to mA‐modulation: 1) patient size adaptation, 2) z‐axis modulation to account for differences in patient attenuation along the z‐axis, and 3) on‐the‐fly angular modulation to account for variations in patient attenuation in the lateral and AP dimensions.

When CARE Dose4D is enabled, the mA at each z‐axis location is calculated from the topogram attenuation map. The mA required to maintain image quality at each z‐axis location for abdomen examinations is determined by calculating the diameter of a cylindrical phantom (with reference tissue‐bone‐air mixture) that would equal the attenuation of the patient at each z‐axis location of the topogram. Based on the difference in diameter between the patient‐equivalent phantom (from the patient topogram) and a reference phantom diameter, the nominal mA is calculated by the CARE Dose4D algorithm.^(6)^ The importance of patient position during localizer acquisition has been described elsewhere^(3)^ but is critical to the success of scans utilizing mA‐modulation. The factors that affect the calculated mA are described below.

#### C.1 CARE Dose4D quality reference mAs

The nominal effective mAs (mA×rotation time÷pitch) for a standard adult reference patient are defined by the quality reference mAs (QRM) parameter. If the measured attenuation is less than that of the reference patient, mA will decrease to maintain image quality without using unnecessary radiation. If the calculated attenuation is higher than that of the reference patient, mA will increase to maintain image quality.

#### C.2 CARE Dose4D strength

The mA correction factor for small and large patients is determined by the CARE Dose4D Strength (S). There are five CARE Dose4D strength settings: 1) very weak, 2) weak, 3) average, 4) strong, and 5) very strong.

The operator's manual^(6)^ provides graphs of mAs modulation versus reference phantom diameter for all CARE Dose4D strengths. Based on the shape of the CARE Dose4D curves and the description of individual CARE Dose4D settings provided in the operator's manual, it was possible to derive the CARE Dose4D algorithm as having the form in the Eq. (1) below:

(1) $$\frac{Effective\;mAs}{Quality\;\text{Ref}erence\;mAs}=e^{(D-D_{\text{Ref}})\cdot S}$$

where D=calculated patient‐equivalent diameter, $D_{ref}=\text{diameter of the reference phantom}$, and S=CARE Dose4D strength. The S values of 0.10, 0.06, and 0.17 were derived to match the curves in the operator's manual for CARE Dose4D strengths of average, weak, and constant noise, respectively. The constant noise curve is shown for illustrative purposes and is not an option in the software. Since QRM is defined as the nominal effective mAs for the reference patient, and both CTDI and SSDE are linear with effective mAs, it is possible to modify Eq. (1) in terms of CTDI and SSDE:

(2) $$\frac{CTDI(D)}{CTDI(D_{\text{Ref}})}=e^{(D-D_{Ref})\cdot S}$$

(3) $$\frac{SSDE(D)}{SSDE(D_{\text{Ref}})}=e^{(D-D_{\text{Ref}})\cdot S}$$

**Figure 3:**
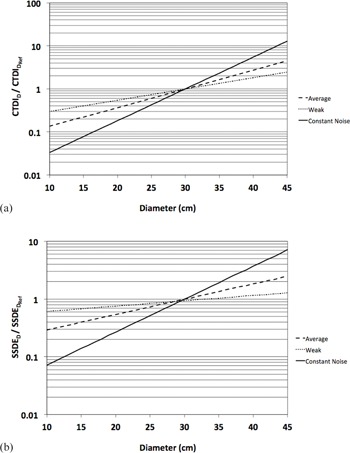
Derived model of CARE Dose4D plotted by normalized (a) CTDI [Eq. (2)] and (b) SSDE [Eq. (3)] for a 30 cm diameter standard adult phantom. Available CARE Dose4D strengths of average and weak are plotted along with a theoretical strength to maintain constant noise.

The reference patient diameter ($D_{\text{Ref}}$) was assumed to be 30 cm since this corresponded to a BW of 34 cm, consistent with the reference adult BW used by Goske et al.^(1)^ (2), (3) are plotted in Figs. 3(a) and 3(b), respectively.

### D. CARE kV overview

In addition to mA‐modulation, CARE kV is designed to automate kV selection with the goal of optimizing the contrast to noise ratio (CNR) for a given diagnostic task. Principles of this model are explained elsewhere;^(7)^ however, we were unable to find detailed information in the Siemens operator's manual. Within the protocol management tool, it is possible to set kV limits for individual protocols. It is also possible to set CARE kV to “Semi” mode where the user manually selects the kV.

#### D.1 CARE kV dose optimizer setting

The Dose Optimizer setting is a slider bar within CARE kV settings with integer values of 0 through 11. The slider bar defines the level of subject contrast for a diagnostic task, from the non‐contrast case with poor structure contrast (0) to the high‐contrast case (11) such as in cardiac exams where iodine is injected to visualize vessels. The principle behind this slider bar is that selecting a low kV will help improve image contrast when there is injected iodine. In these “high contrast” cases, increased noise can be tolerated while maintaining a constant CNR and a lower kV is preferred. In cases with poor subject contrast, there is no advantage to using a lower kV since the noise level is the main determinant of image quality and low kV is not preferred.

### E. CT scan protocols

To start, a baseline “standard” CT protocol was built off a Siemens default abdomen protocol that came loaded on the scanner at installation. This protocol matched the quality reference mAs in the AAPM CT Protocol Working Group's Recommendations for Adult Abdomen/Pelvis examinations^(8)^ and was used in our hospital prior to this study. Two parameters were modified from the default protocol: 1) detector configuration was changed from 16×1.2 mm to 40×0.6 mm for all protocols, and 2) the pitch was changed from 0.6 to 1.3 to scan the newborn, 1‐, 5‐, and 10‐year‐old phantoms. These changes mirrored our clinical protocol and were made to improve the image quality of multiplanar reformats and reduce motion artifacts, respectively.

Each phantom was scanned with all combinations of CARE Dose4D and CARE kV settings summarized in Table 2. This allowed us to empirically model the effect of each parameter on SSDE for each phantom size.

When CARE kV was employed, the lower and upper kV ranges were set to 80 kV and 140 kV, respectively. QRM settings were 150 and 200. The dose optimizer settings were 7 (default) and 3 (lower contrast), denoted by S7 and S3 respectively. CARE Dose4D settings utilized were: very weak, weak, and average. The strong and very strong settings were excluded based on preliminary testing that resulted in SSDE values below the “average” strength and far below target values for pediatric patients and were, therefore, judged to be inappropriate for small patients. All phantoms were scanned using “Semi” mode with manual settings of 80 and 100 kV

**Table 2:** CARE Dose 4D and CARE kV scanning parameters used to develop empirical model.

*Quality Reference mAs*	*Dose Optimizer Setting*	*CARE Dose4D Strength*	*kV Mode*
150	7	Average/Weak/Very weak	CARE kV
150	3	Average/Weak/Very weak	CARE kV
200	7	Average/Weak/Very weak	CARE kV
150	7	Average/Weak/Very weak	Semi 100
150	7	Average/Weak/Very weak	Semi 80^a^

^a^ Only phantoms with BW ≤ 28.35 cm (15 year old phantom) were scanned.

Note: A pitch of 1.3 was used for phantoms <15 years of age; a 0.6 pitch was used for phantoms ≥15 years of age.

### F. Establishment of target radiation dose levels

**Figure 4:**
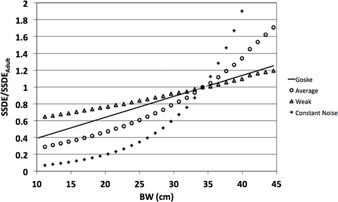
Linear fit of SSDE vs. body width (BW) published by Goske et al.^(1)^ and three CARE Dose4D strengths calculated from Eq. (3).

The target SSDE curve used in this study was based on data published by Goske et al.^(1)^ as part of the Quality Improvement Registry for CT Scans in Children (QuIRCC), a consortium of six pediatric hospitals that contributed data to the American College of Radiology (ACR) Dose Index Registry. The linear fit of SSDE versus body width (BW) presented by Goske and colleagues is shown below:

(4) $$SSDE=(0.14+0.025\times BW)\times SSDE_{Adult}$$

where ${\text{SSDE}}_{\text{Adult}}$ is the SSDE calculated for a standard adult patient with BW=34 cm. Using Eq. (4), we can define the target SSDE for a wide range of patient sizes (i.e., BW) if we know the SSDE for a standard adult. The target SSDE curve [Eq. (4)] is shown in Fig. 4 plotted with CARE Dose4D curves calculated from Eq. (3). For the purpose of creating Fig. 4, patient diameter in Eq. (1) was converted to equivalent BW for each phantom size to match the size metric (i.e., x‐axis values) used by Goske et al. In addition, the CARE Dose4D reference patient was set to a 34 cm BW to match the reference adult size in the Goske model. In order to create the CARE Dose4D models with respect to BW as opposed to diameter (as specified by Siemens), we developed a conversion table based on the BW and effective diameter of our anthropomorphic phantoms. Thus, we were able to plot the Goske model and the CARE Dose4D curves against BW shown in Fig. 4 with a reference patient of 34 cm BW, as used in the Goske study. This is seen in Fig. 4 where all curves converge at BW=34 cm. In our study, ${\text{SSDE}}_{\text{Adult}}$ was the measured SSDE for the medium adult phantom (BW=34 cm) scanned with the default protocol.

For the purpose of illustrating the process of building CT protocols when absolute SSDE values are known, we used 25th percentile values of the DRRs published by Goske et al.^(1)^ These were taken as reasonable target dose levels even though it is defined as the minimum dose required to produce a diagnostic image. However, the Goske study did not consider iterative reconstruction (IR) algorithms and we feel the recent wide adoption of IR with potential for dose reduction^9^, ^10^ makes this level an appropriate target for illustration purposes.

## III. RESULTS

The SSDE for each phantom size and all CARE Dose4D and CARE kV settings are plotted in 5, 6. The target SSDE curve (solid line) is shown for comparison with each configuration. The results are also summarized in Table 3.

**Figure 5:**
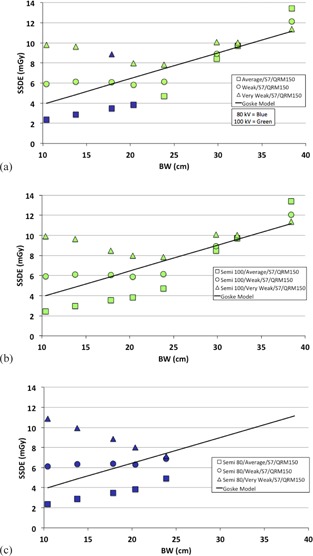
Measured SSDE vs. body width (BW) for very weak, weak, and average strengths of CARE Dose4D and CARE kV setting in (a) Auto mode, (b) Semi mode at 100 kV, and (c) Semi mode at 80 kV. Nominal SSDE target curve published by Goske et al.^(1)^ is plotted for comparison.

Figure 5 shows the effect of changing the Care Dose4D strength with CARE kV in Auto mode, “Semi” mode at 100 kV and “Semi” mode at 80 kV. Results are only plotted up to 23.85 cm for the 80 kV setting due to tube peaking in larger patients. Siemens default values were set for the dose optimizer (S7) and QRM (150). There was large variation in SSDE between the average, weak, and very weak CARE Dose4D strength at BW<30 cm. The ratios ${\text{SSDE}}_{\text{weak}}/{\text{SSDE}}_{\text{avg}}$ and ${\text{SSDE}}_{\text{very}}\;\text{weak}/{\text{SSDE}}_{\text{avg}}$ for a 10.5 cm (newborn) phantom were 2.5 (5.9/2.4) and 3.8 (9.1/2.4) respectively. Selection of the “Semi” mode resulted in SSDE values similar to Auto mode for all phantom sizes, but the kV was set to the user‐defined kV.

**Figure 6:**
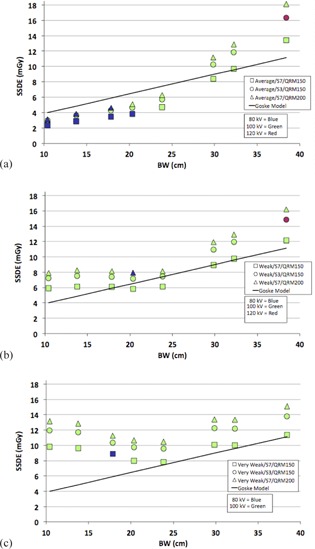
Measured SSDE vs. body width (BW) for two different dose optimizer settings (S7 and S3), two different quality reference mAs settings (150 and 200) with CARE kV setting of “Auto” for CARE Dose 4D strengths of average (a), weak (b), and very weak (c).

The effect of changing the QRM and Dose Optimizer settings are shown in Fig. 6 for CARE Dose4D strengths of average, weak, and very weak. Changing the QRM affected the SSDE by a factor equal to the ratio of ${\text{QRM}}_{\text{new}}/{\text{QRM}}_{\text{old}}$ for all phantom sizes and is consistent with the behavior predicted by Eq. (1).

The effect of changing the Dose Optimizer from S7 to S3 was more complicated and was a function of curve strength. In the case of the weak and very weak curves, which used 100 kV at S7 (Fig. 5(a)) for all phantom sizes (with the exception of one anomalous data point at BW = 17.85 cm with the very weak curve), the kV selection was not affected by changing the dose optimizer from S7 to S3. In this case, the ratio ${\text{SSDE}}_{S3}/{\text{SSDE}}_{S7}=1.22$ for all phantoms. For the average curve, where 80 kV was used for phantom sizes <23.85 cm with S7 (Fig. 4(a)), changing the dose optimizer setting from S7 to S3 resulted in a kV selection of 100 for these phantoms sizes. In this case, the ratio SSDE_S3_/SSDE_S7_ was 1.21 (4.64/3.83), 1.23 (4.28/3.47), 1.26 (3.64/2.88), and 1.28 (3.00/2.35) for BW=10.42,13.78,17.85, and 20.36 cm, respectively.

**Table 3:** Summary of effects of CARE Dose4D and CARE kV scanning parameters as compared with top row default protocol.

*CARE Dose 4D Strength*	*Quality Reference mAs*	*Dose Optimizer*	*CARE kV*	*SSDE* BW<32 cm	*SSDE* BW>32 cm	*Scan kV*
Average	150	7	Auto			
Weak^e^	150	7	Auto	Increase^a^	Decrease^a^	100^b^
Very weak^e^	150	7	Auto	Increase^a^	Decrease^a^	100^c^
Average	200^e^	7	Auto	Increase (133%)	Increase (133%)	80/100^d^
Average	150	3^e^	Auto	Increase	Increase	100^b^
Average	150	7	Semi^e^ 80	None	None	80
Average	150	7	Semi^e^ 100	None	None	100

^a^ Size dependent.

^b^ All phantom sizes.

^c^ Exception: 80 kV @ body width (BW) = 18 cm.

^d^ <18 cm, 80 kV; ≥18 cm, 100 kV

^e^ Different parameters from the default protocol.

## IV. DISCUSSION

We have assumed that SSDE is an appropriate surrogate for image quality in the context of designing CT protocols. A study by Larson et al.^(11)^ showed inter‐reviewer variability within a single pediatric radiology department was very large when scoring the same image. From this, we thought it reasonable to assume that SSDE is a reasonable surrogate for image quality since even more direct metrics of image quality (e.g., image noise) are limited by radiologist preference.

### A. Empirical model of CARE Dose4D and CARE kV

The results of this phantom study allowed us to develop an empirical model of CARE Dose4D and CARE kV for a range of phantoms sizes. From this model, it was possible create simulated protocols that achieve target kV and SSDE levels across a full range of patient sizes.

When the QRM and Dose Optimizer settings were not modified from the default protocol, SSDE converged approximately at the medium adult phantom size of approximately 32.3 cm lateral dimension (Fig. 5(a)), in agreement with the nominal operation of CARE Dose4D where the dose for all CARE Dose4D strengths converge to the standard adult reference patient.

The effect of QRM on SSDE (Fig. 6) across all patient sizes matched that predicted by Eq. (1). The effect of QRM is to simply change the baseline dose (i.e., the dose for all patient sizes) by a fixed ratio. For example: by changing the QRM from 150 to 200 (Table 3), the ratio of ${\text{SSDE}}_{200}/{\text{SSDE}}_{150}=1.34$ or approximately the ratio of the QRM values (i.e., 200/150=1.33).

The effect of using “Semi” mode in the CARE kV setting (Figs. 5(b) and (c)) was also consistent with the definitions in the operator's manual and our predicted behavior. Employing the “Semi” mode permitted manual selection of kV without affecting baseline dose or CARE Dose4D curve shape. This finding, of “Semi” mode only affecting dose without affecting curve shape, is important as it allows the user to customize protocols by defining a desired kV and CARE Dose4D strength independently. Therefore, size‐specific protocols that achieve target SSDE levels with user‐defined kV can be programmed using “Semi” mode.

The effect of changing the Dose Optimizer was consistent with the understanding of this parameter as essentially increasing noise for higher slider settings. However, the ratio ${\text{SSDE}}_{3}/{\text{SSDE}}_{S7}∼1.22$ when the kV is unchanged was previously unknown and is an important piece of information when designing CT protocols. Since the effect of changing the Dose Optimizer can also be accomplished by changing the QRM (by a ratio of 1.22), but with more fine‐tuning in the case of the QRM parameter (since QRM is defined in 1 mAs increments), it may make sense to eliminate the Dose Optimizer setting as a variable in protocol design. For this reason, the default S7 value was used when defining our size‐specific protocols in the next section.

The most important finding from this study was that changing CARE Dose4D strength had a direct effect on kV selection when CARE kV was in “Auto” mode. In particular, the weak and very weak curves both use 100 kV across all patient sizes and do not use 80 kVp for even the smallest patients. There was a single anomalous result where the very weak curve selected 80 kVp for the 18 cm phantom size, which could not be explained since 80 kVp was not selected for smaller patients. This anomalous behavior was nonetheless reproducible. The effect of CARE Dose4D strength on kV selection has implications for protocol optimization. In our case, we would prefer to use a CARE Dose4D strength of weak, but also take advantage of the higher CNR at 80 kVp. For this reason, our size‐based protocols in the next section use “Semi” mode setting for CARE kV.

The most practical result from this study was that no single CARE Dose4D strength followed the shape of SSDE target model published by Goske et al.^(1)^ for the full range of phantom sizes and it is unlikely to closely match an institution‐specific SSDE target curve. To achieve customized reference levels, we need to design size‐based protocols with size‐specific manual kV and CARE Dose4D parameters.

There were several limitations to our study. First, only a single‐manufacturer AEC algorithm was modeled and this model cannot be applied to other AEC algorithms from different manufacturers. However, methods have been published to match dose across manufacturer platforms^(12)^, therefore, if AEC is designed appropriately for one manufacturer, it can be matched to other scanner models, making our results potentially generalizable. This study used anthropomorphic tissue‐equivalent phantoms to model an AEC algorithm. Deviation of patient morphology and attenuation could result in SSDE values that are different from our phantom results. However, it is not recommended to use human subjects for AEC modeling or protocol optimization due to safety concerns and the wide range in patient size and shape encountered in clinical practice.

### B. Process for protocol optimization

Using CARE Dose4D and CARE kV in a fully automated mode (i.e., only two protocols, one each for adult and pediatric patients with CARE kV turned “ON”) may be a reasonable choice for a large hospital with limited physics support, various scanner models, and a large number of rotating technologists. This approach could potentially eliminate sources of error in protocol selection and is appropriate when a higher degree of customization is not feasible. However, for pediatric–focused facilities with physics support, it is possible to design a set of size‐based protocols that match either published target dose levels^(1)^ or hospital‐specific DRRs in accordance with the ALARA principle. New Joint Commission standards require establishment of diagnostic reference levels (DRLs) and this study presents a method of consistently achieving these DRLs.

The following is an example of the process for building dose‐optimized protocols.

**Figure 7:**
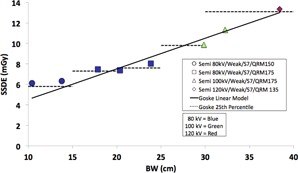
Simulated SSDE vs. body width (BW) for size‐specific CT protocols listed in Table 4.

We chose to use “ Semi” mode for our size‐based protocols. We found that using “ Semi” mode did not change the shape of the CARE Dose4D curves and eliminated one variable. We fixed the Dose Optimizer setting at 7, and instead modified the QRM in our size‐based protocols, which can produce the same effect, thereby eliminating a second variable and potential source of anomalous results. Using the linear relationship between QRM and SSDE, we adjusted QRM to match the SSDE levels defined by the 25th percentile values of the QuIRCC group.^(1)^ The size‐based protocols that accomplish this task are summarized in Table 4 and plotted in Fig. 7, along with the 25th percentile level of published DRRs. Figure 7 demonstrates how protocols using size‐based QRMs and manual kV can achieve target dose levels across all patient sizes while taking advantage of AEC dose saving features.

**Table 4:** Simulated size‐based CT protocols that achieve published SSDE target levels at optimized kV

*Lateral Dimension (cm)*	*Potential Weight Category (kg)*	*Quality Reference mAs*	*Dose Optimizer Setting*	*Care Dose Strength*	*kV (Semi)*
<15	<10	150	7	Weak	80
16–25	11–30	175	7	Weak	80
26–35	31–70	175	7	Weak	100
>36	>71	135	7	Weak	120

It should be noted that the trade‐off in designing size‐based protocols with different CARE Dose4D and CARE kV settings is an increased responsibility on the part of the technologist to properly center the patient,^(3)^ accurately measure the lateral body size from the localizer and, finally, select the correct scanning protocol.

Our process for protocol optimization is:

1. Use lateral patient dimension (measured from PA localizer) for protocol selection;
2. Use “Semi” mode with size‐specific kV (see Table 4);
3. Keep slider position at default value (e.g., S7);
4. Use the CARE Dose4D strength that most closely fits the nominal SSDE vs. patient size curve (e.g., weak);
5. Adjust QRM for size‐based protocols to match target dose levels (see Table 4); and
6. Merge protocols if possible (i.e., if adjacent size categories have identical settings).

## V. CONCLUSIONS

In our study, we developed an empirical model for Siemens CARE Dose4D and CARE kV with respect to SSDE and automatic kV selection for a wide range of phantom sizes. Using this model, optimized protocols can be created based on either published or hospital‐specific dose targets prior to scanning patients. We proposed simulated protocols that match the 25th percentile target level published by Goske et al.^(1)^ Size‐based protocols with customized AEC parameters can be the most reliable method for dose optimization.

## Appendix

Our study identified several limitations of the CARE Dose and CARE kV software.

Specifically, that low kV's were “blocked” when “weak” and “very weak” CARE Dose strengths were enabled. At the time, this limitation forced our facility to develop a work‐around by creating size‐based protocols with manual kV selection utilizing “Semi” mode described in the paper.

Since our study was conducted in June 2014, we have discovered this anomalous result was probably caused by the software version. In more recent versions (VG51B and higher), Siemens appears to have corrected this limitation. Our results remain valid and important, and the software version is clearly stated in the Methods section.
